# Remarks on the Seasonal Incidence of Diarrhœa, and the Part Played by Flies


**Published:** 1903-01-31

**Authors:** J. T. C. Nash


### HOSPITAL CLINICS AND MEDICAL PROGRESS.

### REMARKS ON THE SEASONAL INCIDENCE OF DIARRHŒA, AND THE PART PLAYED BY FLIES.*

By J. T. C. NASH, M.D.EDIN., D.P.H.CANTAB.

ON November 12th, 1902, at a conference of Essex medical officers of health which was convened to discuss infantile mortality in Essex, I recorded my very strong conviction that imperfect methods of disposal of refuse and excreta ranked among the chief causes of infantile mortality, because, apart from the risk of infected dust being blown about by wind, collections of organic filth attracted and bred flies, and in my opinion flies were one of the chief agents in the causation and spread of diarrhœa. We are aware that the diarrhœa mortality curve based upon the records of many years in London, shows a slight rise in June, rapidly increasing during July, until it reaches its summit early in August, after which it again rapidly falls. A careful study of the London diarrhœa curve reveals that in the second week in June the rise commences from the fairly dead level of about 12 deaths a week which is the average obtaining from December to the beginning of June. In the third week of June the number of deaths has reached 50 in the week. From this time the rate of increase becomes very rapid so that the first week in July sees the number of deaths raised to 100, the second week in July to 150, the third week to 200. For about a fortnight the figure is somewhere above 200 deaths per week. Then it begins to fall, reaching 160 about the end of August, 100 by the middle of September, 50 by the beginning of October, and then the rate of decrease is more gradual until it reaches the dead level of about 12 deaths a week on an average towards the end of November. Ballard's inquiry into the causation of diarrhœa and his conclusions are well known. The valuable charts he made for London and many other towns showing week by week for many years the earth temperatures at a depth of 1 foot and 4 feet from the surface, and the diarrhœal mortality of the corresponding weeks led him to formulate the conclusions that the summer rise of diarrhœal mortality does not commence until the mean temperature recorded by the four-feet earth thermometer has attained somewhere about 56° Fahr., that the diarrhœa maximum mortality coincides with the mean weekly maximum of the four-feet earth temperature and that the slower fall of the earth temperature coincided with the slower decline as compared with the rise of the diarrhœal mortality.

All must admit the coincidence of the earth temperature and the diarrhœa mortality, and no doubt an indirect causal relation obtains; but I join issue with Dr. Ballard in his conclusions that the essential cause of diarrhœa is a specific micro-organism residing in the superficial layer of the earth whose vital manifestations are dependent upon conditions of season, etc. I am prepared to admit that the essential cause is bacterial in nature, but I think all evidence goes to show that there is no one specific bacterium of diarrhœa; if instead of micro-organism, we merely say an organism whose vital manifestations are dependent upon conditions of season, we shall, I think, be nearer the truth. I suggest that this organism is the common housefly which, beginning to make its appearance in June, becomes a veritable pest during July and the early part of August. Towards the end of August its existence tends to become a mere sexual one, and it rapidly decreases in numbers thereafter, though occasional specimens may be found into November. This creature is often termed the harmless fly. I certainly give it the first place as a pathogenic agent during the summer months. It is a useful scavenger if kept in its place, but when allowed to fly straight from the dung or refuse heap to commit suicide in the milk bowl, or alight on the lips of a sleeping infant, or walk over meat and other articles of food on the table, I repeat that I consider it the most active pathogenic agent during the summer and the principal cause of summer diarrhœa. It is in milk that it proves most dangerous, for it falls into milk bearing on its body and legs such germs as bacillus coli, proteus vulgaris, bacillus enteriditis (Gärtner), proteus zenkeri, streptococci, staphylococci, and other pathogenic germs. The bacteria I have mentioned have actually been isolated from flies taken from a refuse heap and examined by Mr. Foulerton at the Middlesex Hospital. By the aid of flies the Eberth-Gaffky and diphtheria bacilli may easily be conveyed to milk.

In milk bacteria find an ideal pabulum and multiply at a most astonishing rate, particularly when the atmospheric temperature is high. This has been shown by various observers, and quite recently at the December meeting of this Society was emphasised by Professor Delépine in his valuable contribution on "The bearing of Outbreaks of Food Poisoning upon the Etiology of Summer Diarrhœa." This contribution is of the highest scientific importance, and I think most will agree that in view of the number and nature of Professor Delépine's observations he is justified in his general conclusions as to the chief cause of epidemic diarrhœa being fœcal contamination of milk at the farm or in transit. All sanatarians will cordially endorse the preventive measures he mentions; indeed, those of us who are medical Officers of Health already strive to secure this cleanliness of dairy premises, cowsheds, milkers, utensils, etc.

Professor Delépine has substantially corroborated what bacteriologists for some years have recognised, viz. that time and temperature are most important factors in the multiplication of bacteria, and consequently in the degree of infectivity of milk.

(*To be Continued.*)

* A contribution to the discussion on the Etiology of Summer Diarrhœa, at the meeting of the Epidemiological Society of London, January 16th, 1903.

